# Molecular dynamics identifies semi-rigid domains in the PD-1 checkpoint receptor bound to its natural ligand PD-L1

**DOI:** 10.3389/fbioe.2022.838129

**Published:** 2022-10-06

**Authors:** Michael Kenn, Rudolf Karch, Lisa Tomasiak, Michael Cibena, Georg Pfeiler, Heinz Koelbl, Wolfgang Schreiner

**Affiliations:** ^1^ Institute for Biosimulation and Bioinformatics, Center for Medical Statistics, Informatics and Intelligent Systems, Medical University of Vienna, Vienna, Austria; ^2^ Division of General Gynecology and Gynecologic Oncology, Department of Obstetrics and Gynecology, Medical University of Vienna, Vienna, Austria

**Keywords:** molecular dynamics, checkpoint inhibitor, immune therapy, oncology, drug design, cluster analysis

## Abstract

Cells in danger of being erroneously attacked by leucocytes express PD-L1 on their surface. These cells activate PD-1 on attacking leucocytes and send them to death, thus curbing erroneous, autoimmune attack. Unfortunately, cancer cells exploit this mechanism: By expressing PD-L1, they guard themselves against leucocyte attack and thereby evade immune clearance. Checkpoint inhibitors are drugs which re-enable immune clearance of cancer cells by blocking the binding of PD-L1 to PD-1 receptors. It is therefore of utmost interest to investigate these binding mechanisms. We use three 600 ns all-atom molecular dynamics simulations to scrutinize molecular motions of PD-1 with its binding partner, the natural ligand PD-L1. Usually, atomic motion patterns are evaluated against whole molecules as a reference, disregarding that such a reference is a dynamic entity by itself, thus degrading stability of the reference. As a remedy, we identify semi-rigid domains, lending themselves as more stable and reliable reference frames against which even minute differences in molecular motion can be quantified precisely. We propose an unsupervised three-step procedure. In previous work of our group and others, minute differences in motion patterns proved decisive for differences in function. Here, several highly reliable frames of reference are established for future investigations based on molecular motion.

## 1 Introduction

### 1.1 Medical background and clinical significance

Immune system T-cells detect cancer cells as they develop, and normally kill them (Smith-Garvin et al., 2009). However, some cancer cells have developed mechanisms to escape this important, immune–mediated clearance (Chen and Mellman, 2013) as follows: T-cells present a suicide tool (PD-1) on their surface. In healthy individuals, this tool is activated (by PD-L1) only if a T-cell should erroneously attack healthy tissue. PD-1 is therefore called an “immune-checkpoint”.

However, some cancer cells also express PD-L1 on their surface. They exploit the above checkpoint mechanism, abusively activate the immune checkpoint molecules (Dong et al., 2002) and thereby escape destruction. By increased expression of PD-L1 and/or the release of immunosuppressive factors cancer cells may survive even in a “hot”, immune-cell enriched surrounding.

Checkpoint inhibitors are drugs blocking the binding between PD-1 and its natural ligand, PD-L1. Clinical trials have proved their efficacy (Brahmer et al., 2012; Kwa and Adams, 2018). More recently a phase III trial in metastatic triple negative breast cancer patients showed a distinct improvement in progression-free survival and overall survival (Brahmer et al., 2018). This demonstrates the significance of the target (PD-1) being expressed when a PD-L1 antibody is used (Schmid et al., 2018; Cortés et al., 2019).

In order to further improve these promising therapies, a better understanding of the molecular mechanism of the PD-1 receptor is necessary.

### 1.2 Rationale for multi-level clustering

To evaluate minute movements within molecular dynamics trajectories, all frames need to be fitted to a certain intramolecular region (i.e. domain) at a reference frame (point in time). Such a fitting domain should not significantly deform itself over time (along a trajectory), in order to serve as a stable reference against which very small and intricate movement patterns outside this domain can be detected.

In previous work, domains for fitting were usually selected manually, based on secondary structure, such as beta-strands, beta-sheets or alpha helices. We detect such stable regions in an unsupervised procedure from the computed dynamics itself. In particular for example, if parts of beta-strands participate in the binding mechanics to be evaluated, they should not at the same time be part of the domain to which fitting is performed.

A most direct approach would be clustering according to small changes in distance between pairs of atoms over the whole trajectory. However, it is known that molecular systems tend to switch between metastable states, each of which may pertain over considerable parts of the simulation. During such a metastable state, some pairs of atoms might remain in close vicinity, with little variation of their distances. For example, atoms in some loop, which assumes a certain conformation characteristic for this and only this meta-state. Clustering only during this meta-state would send these pairs into the same cluster. However, as the system switches to another meta-state, the very same pairs of atoms could be detached from each other, become members of different neighborhoods and end up in different clusters if clustering would be performed only over this second meta-state. In consequence, one single pass of clustering over the whole trajectory might particularly conceal minute patterns of motion, being of focal interest. Separate clustering of segments of a trajectory is likely to take account of such minute differences between meta-states and exclude these regions from semi-rigid domains to be obtained.

Deriving rigidity directly and unsupervised from the simulation is considered a promising advantage and basis for future MD-studies.

### 1.3 Molecular structures

**FIGURE 1:**
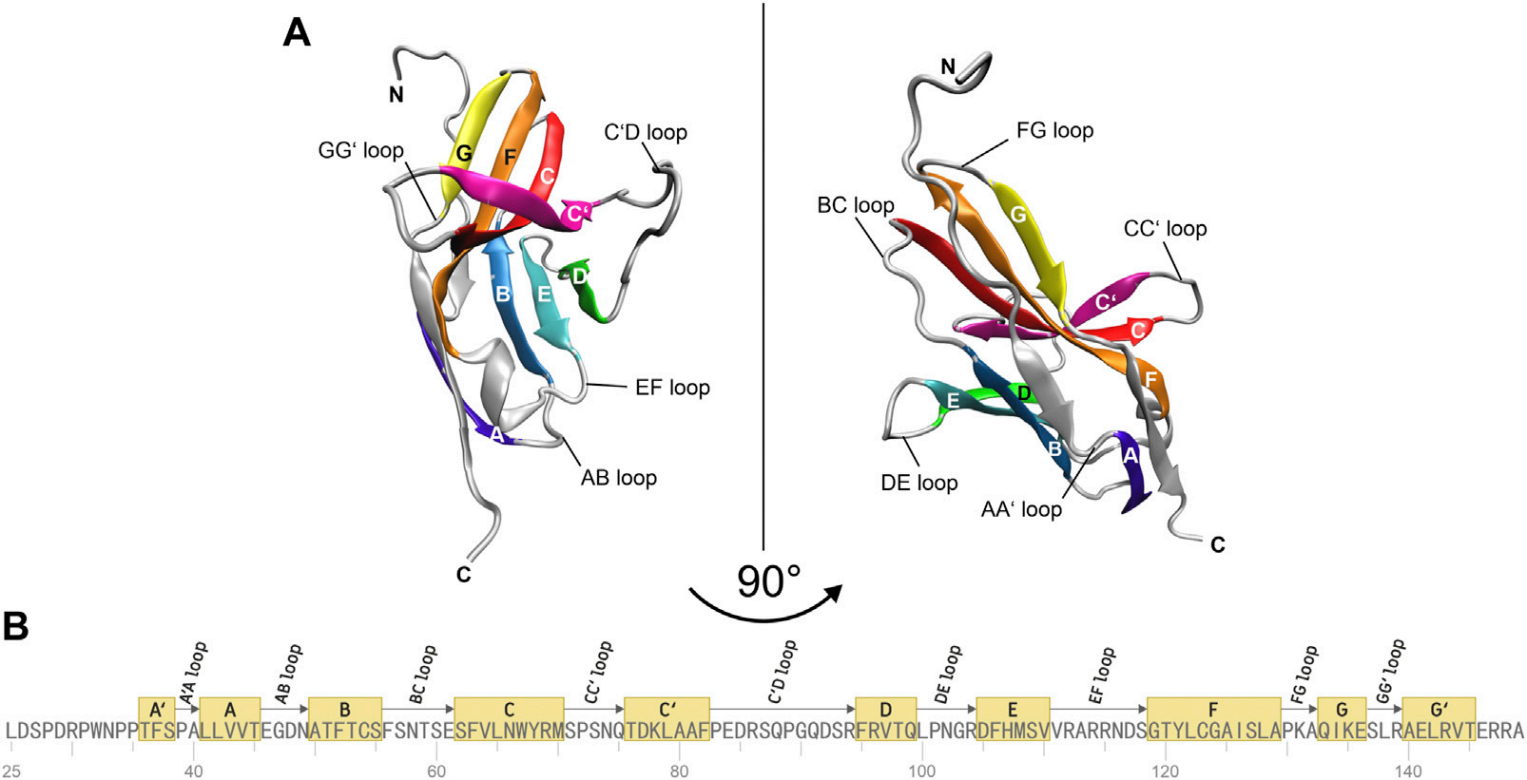
Molecular structure of immune checkpoint molecule PD-1. **(A)** Cartoon representation of the extracellular domain of PD-1. A two-layer β sandwich is formed by two β sheets GFCC’ (colored yellow, orange, red, magenta) and ABED (colored violet, blue, cyan, green) with loops connecting the respective β strands (colored silver). **(B)** Sequence of the residues of PD-1. The β strands of the protein are depicted as yellow boxes and the connecting loops as arrows. The figures were prepared using VMD version 1.9.3 (Humphrey et al., 1996).

**FIGURE 2:**
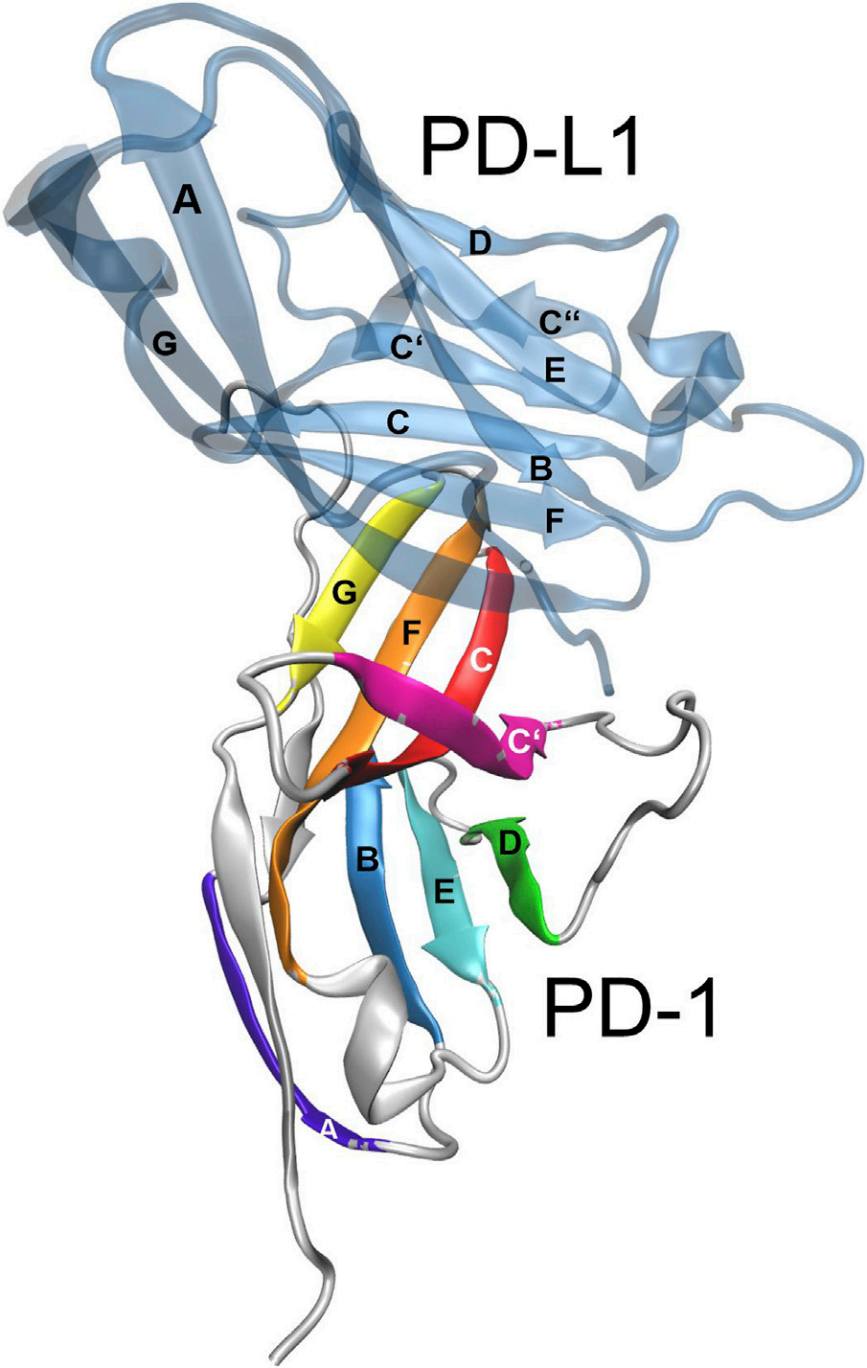
Immune checkpoint molecule PD-1 and binding partners. Cartoon representation of the extracellular domain of PD-1 bound to the endogenous ligand PD-L1 (transparent blue). The figure was prepared using VMD version 1.9.3 (Humphrey et al., 1996).

The molecular structure of the immune checkpoint PD-1 is shown in Figure 1, generated with VMD (Humphrey et al., 1996; Hsin et al., 2008; Cross et al., 2009) from PDB (Burley, 2013) entry 4ZQK (Zak et al., 2017). Since 4ZQK does not contain the complete structure of PD-1, we have modelled the missing parts *in silico* already in our previous work (Roither et al., 2021). The immune checkpoint receptor, PD-1, consists of several beta strands in tight mutual binding and respective loops in between, see Table 1. These loops protrude loosely from a rather compact beta core and offer versatile modes of interaction and binding. In particular, the residues 70 to 77, comprising the CC′-loop, are crucial for interaction with the natural ligand PD-L1 (Kundapura and Ramagopal, 2019), see Figure 2. Details of this interaction have been investigated experimentally by Zak (Zak et al., 2015) and in molecular dynamics studies by Liu (Liu et al., 2017) and our group (Roither et al., 2020; Tomasiak et al., 2020; Roither et al., 2021; Tomasiak et al., 2021).

**TABLE 1:** Residues and secondary structure of PD-1. The assignment of strands and loops was chosen according to the classification of the Protein Feature View applet available within the 4ZQK record of the PDB. The domains were named following canonical Ig-strand designations (Zak et al., 2015). ResID_S_ and ResID_E_ indicate the starting and the ending residue ID of the according domain within chain B of 4ZQK. Res#_S_ and Res#_E_ indicate the starting and the ending residue number of a domain (continuous numbering for the whole complex in the respective PDB file).

Domain	ResID_S_	ResID_E_	Amino acid sequence	4ZQK	
				Res#_S_	Res#_E_
NtermA′ loop	25	35	LDSPDRPWNPP	116	126
A′ strand	36	38	TFS	127	129
A'A loop	39	40	PA	130	131
A strand	41	45	LLVVT	132	136
AB loop	46	49	EGDN	137	140
B strand	50	55	NATFTCS	141	146
BC loop	56	61	FSNTSE	147	152
C strand	62	70	SFVLNWYRM	153	161
CC′ loop	71	75	SPSNQ	162	166
C′ strand	76	82	TDKLAAF	167	173
C'D loop	83	94	PEDRSQPGQDSR	174	185
D strand	95	99	FRVTQ	186	190
DE loop	100	104	LPNGR	191	195
E strand	105	110	DFHMSV	196	201
EF loop	111	118	VRRRNDS	202	209
F strand	119	129	GTYLCGAISLA	210	220
FG loop	130	132	PKA	221	223
G strand	133	136	QIKE	224	227
GG′ loop	137	139	SLR	228	230
G′ strand	140	145	AELRVT	231	236
G’rest loop	146	149	ERRA	237	240
PD-L1 binding domain	70	77	MSPSNQTD	161	168
Pembrolizumab binding domain	74	99	NQTDKLAAFPEDRSQPGQDCRFRVTQ	165	190
NtermA′ loop	25	35	LDSPDRPWNPP	116	126

In the present work we draw on previous experience with the same system (Roither et al., 2020; Roither et al., 2021) but focus on unsupervised clustering, using a very efficient algorithm (Kenn et al., 2016) previously developed for MHC-molecules and T-cell receptors (Kenn et al., 2014).

## 2 Materials and methods

Molecular preparation and technical details of the molecular dynamics (MD) simulation have already been reported (Tomasiak et al., 2021). In Sections 2.1, 2.2, we briefly recapitulate essential points for completeness. The remaining subchapters Sections 2.3.1–2.3.5 refer to evaluation methods specific for this work.

### 2.1 Preparation of molecular complexes

Structural data for MD simulations were downloaded from the protein data bank (PDB, https://www.rcsb.org/) using the following entries: PDB-ID 4ZQK for the PD-1/PD-L1 system (resolution: 2.45 Å) (Zak et al., 2015) and PDB-ID 5GGS for the PD-1/pembrolizumab Fab fragment complex (resolution: 2.0 Å) (Lee et al., 2016). Missing residues in the crystal structure of the endogenous ligand PD-L1 in complex with the extracellular domain of PD-1 (PDB-ID 4ZQK), were added from the PD-1/pembrolizumab system (PDB-ID 5GGS), the N loop was taken from the PD-1/nivolumab system (PDB-ID 5WT9), see Roither et al. (Roither et al., 2020) for further preprocessing details. For determining the protonation states at pH 7.0 the H++ Server was used (http://biophysics.cs.vt.edu/) (Gordon et al., 2005). The assignment of strands, sheets, and loops was made following the classification of the Protein Feature View applet available within the 4ZQK record of the PDB (see Figure 1B).

### 2.2 All-atom molecular dynamics

As described previously (Tomasiak et al., 2021) all-atom MD simulations were performed with GROMACS 2021.2 (Hess et al., 2008), using the Amber99sb-ildn force field (Lindorff-Larsen et al., 2010) and an explicit water model. For the simulation box a rhombic dodecahedron was chosen with a minimum distance of 2 nm between the respective molecules and the box boundaries. The PD-1/PD-L1 complex consists of 4099 atoms and 240 residues, and the complex was solvated in TIP3P water (Jorgensen et al., 1983). Solute molecules were replaced by sodium and chloride ions to reach a physiological salt concentration of 0.15 mol/L.

For the energy minimization the method of steepest-descent was chosen. Before production runs the systems were equilibrated at NVT and NPT for 100 ps (time step 2 fs) each. In the NVT equilibration run the temperature was set to 310 K using a Berendsen-thermostat (Berendsen et al., 1984) with a time constant of 0.1 ps and position restraint MD. Equilibration in NPT ensembles was performed under the control of a Berendsen-barostat (Berendsen et al., 1984) set to 1 bar with a time constant of 1.0 ps.

All independent production runs had a simulation time of 600 ns with a time step of 2 fs using the LINCS algorithm (Hess, 2008) for constraining bonds to hydrogen atoms. For the van der Waals interactions a single cut-off of 1.47 nm was used and a cut-off distance of 1.4 nm for the short-range neighbor list in the Verlet scheme (Verlet, 1967) for neighborhood search. For electrostatic interactions the particle-mesh Ewald (PME) algorithm (Darden et al., 1993) was applied with a cut-off of 1.4 nm. Temperature coupling was done with the velocity-rescaling algorithm (Bussi et al., 2007) at a temperature of 310 K and for pressure coupling at 1 bar the Parrinello Rahman algorithm (Parrinello and Rahman, 1981) was used with a time constant of 2 ps. 30000 frames for each run were obtained by saving coordinates, velocities, forces, and energies every 20 ps to a trajectory file. Three independent 600 ns MD simulations with different initial velocities were carried out for each system, summing up to a total simulation time of 600 ns * 3 = 1.8 μs.

Prior to the evaluation, all frames of each given trajectory were fitted to the first frame of the trajectory, according to minimum root mean square deviation (RMSD) at time *t*. In mathematical terms, the Cartesian coordinates $x_{i}$ of all atoms *i* were translated and rotated towards minimum RMSD of the backbone within β-strands and α-helices:

$$RMSD\left(t\right)={\left[\frac{1}{N_{bb}}\overset{N_{bb}}{\underset{i=1}{\sum }}{\left\|x_{i}\left(t\right)-x_{i}\left(0\right)\right\|}^{2}\right]}^{1/2}\to \mathrm{Min}$$ (1)

where **x**_i_(*t*) is the position of atom *i* at time *t*. For the precise regions of secondary structure elements (β-strands and α-helices), see Tables 1, 2. *N*_bb_ is the total number of backbone atoms (N, C_α_, C_O_) contained therein. Finally, the first 100 ns of each trajectory were discarded to get rid of initial phase trends, leaving 500 ns with *N*_t_ = 25000 frames for each trajectory to be further analyzed.

**TABLE 2:** Residues and secondary structure of PD-L1. The assignment of strands, loops and helices was chosen according to the classification of the Protein Feature View applet available within the 4ZQK record of the PDB protein data bank. The domains were named following canonical Ig-strand designations (Zak et al., 2015). Res#_S_ and Res#_E_ indicate the starting and the ending residue number of a domain (continuous numbering for the whole complex in the respective PDB file).

Domain	ResID_S_	ResID_E_	Amino acid sequence	Res#_S_	Res#_E_
NtermA loop	18	26	AFTVTVPKD	1	9
A strand	27	31	LYVVE	10	14
AB loop	32	35	YGSN	15	18
B strand	36	41	MTIECK	19	24
BH1 loop	42	48	FPVEKQL	25	31
Helix1	49	52	DLAA	32	35
H1C loop	53	53	L	36	36
C strand	54	59	IVYWEM	37	42
CC′ loop	60	61	ED	43	44
C′ strand	62	68	KNIIQFV	45	51
C'C″ loop	69	70	HG	52	53
C″ strand	71	72	EE	54	55
C″H2 loop	73	73	D	56	56
Helix2	74	82	LKVQHSSYR	57	65
H2D loop	83	84	QR	66	67
D strand	85	87	ARL	68	70
DH3 loop	88	88	L	71	71
Helix3	89	94	KDQLSL	72	77
H3E loop	95	95	G	78	78
E strand	96	101	NAALQI	79	84
EH4 loop	102	104	TDV	85	87
Helix 4	105	109	KLQDA	88	92
F strand	110	117	GVYRCMIS	93	100
FG loop	118	120	YGG	101	103
G strand	121	131	ADYKRITVKVN	104	114
Grest loop	132	132	A	115	115

### 2.3 Obtaining semi-rigid domains

Semi-rigid domains for a given trajectory were obtained in a two-step process: First, “spatial clustering” was performed by grouping C_α_-atoms showing similar movements into each of the clusters. Evidently, such a clustering does not need to (and will not) yield exactly the same clusters if spatial clustering is performed for different subsections of *N*_f_ frames each (called “segments” in the following) of a trajectory: Some pairs of C_α_s will stay together in a given cluster over many segments, others will not (Kenn et al., 2014). This fact is exploited to perform “time-wise clustering” as a second step, by constructing new clusters from those C_α_s which stay together within spatial clusters across successive segments with maximum fidelity. Such groups of atoms form clusters even more stable over time and are hence termed “semi-rigid domains” (Kenn et al., 2016). The total number of frames used from a trajectory, *N*_t_, is partitioned into *N*_s_ segments, with *N*_t_ = *N*_s_ * *N*_f_. We used *N*_s_ = 500 and *N*_f_ = 50, corresponding to 1 ns per segment and a frame length of 20 ps.

Note that time-wise clustering is a special mode of consensus clustering (Monti et al., 2003), since the same clustering algorithm is applied to different parts of a trajectory and a consensus between these results is finally adopted.

#### 2.3.1 Spatial clustering

**FIGURE 3:**
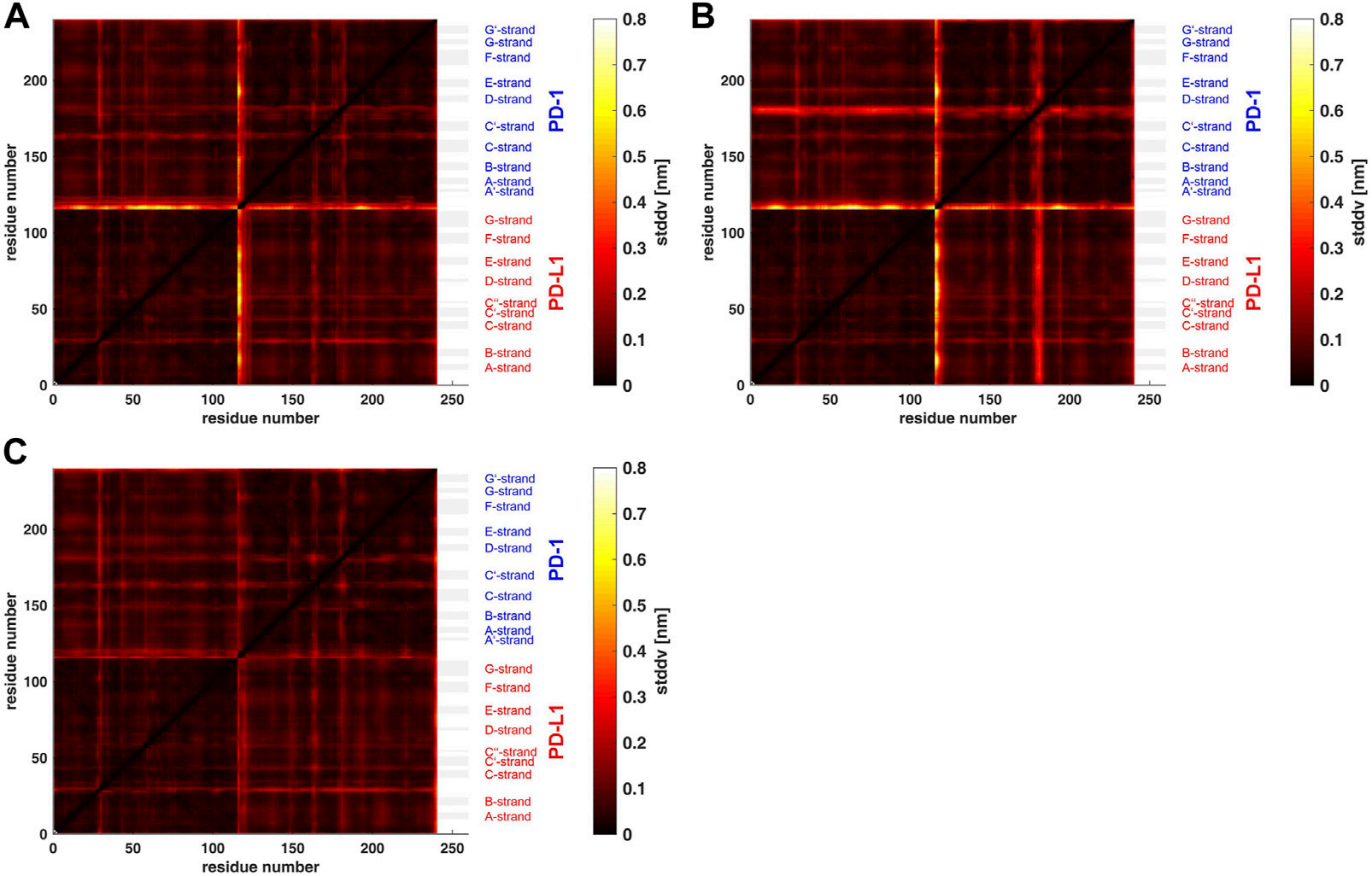
Matrix of standard deviations of atom distances over whole trajectories, shown as scaled color image (SCI). **(A)**: Trajectory *t*_1_ for complex 4ZQK, consisting of receptor PD-1 and PD-L1 as ligand, showing enhanced similarity within two large areas (receptor and ligand, respectively). Note that numbering starts with ligand PD-L1 with residue-ID = 18 (lower left corner), corresponding to residue number *i* = 1 in both axes of the SCI shown. PD-L1 extends over 1 ≤ *i* ≤ 115. The N-terminal end of PD-1 starts with residue-ID = 25 and extends over residues 116 < *i* < 240 towards the right upper corner. Elements of secondary structure are denoted right to the SCI (Zak et al., 2017), with their extensions indicated by horizontal grey shaded bars. Standard deviations *D*_*ij*_ [nm], computed according to Eq. 2, for values see color bar. **(B)**: trajectory *t*_2_. **(C)**: trajectory *t*_3_.

One crucial aspect of collective motion of atoms is captured by the variability (standard deviation) of mutual distances (Kenn et al., 2014), usually termed STDDV. We use it as an approximation for “motional distance” between two C_α_s *i* and *j*, and denote it for brevity by *D*_*ij*_ defined as

$$D_{ij}=\sqrt{\frac{N_{f}}{N_{f}-1}\langle {\left(d_{ij}-\langle d_{ij}\rangle \right)}^{2}\rangle }$$ (2)

where $d_{ij}=\left\|x_{i}-x_{j}\right\|$ is the Euclidean distance in a given (time-wise) frame and 〈〉 denotes averaging over all *N*_f_ frames for which clustering is intended, see Figure 3. Note that distances are not affected by any fitting of the trajectory to a reference frame.

For actual spatial clustering (over segments or over the whole trajectory) we consider C_α_ atoms only and follow the concept of Bernhard and Noé (Bernhard and Noé, 2010). Each $C_{\alpha ,i}$ is assigned a membership in cluster *m*, expressed as a real number $0\leq c_{i,m}\leq 1$ , with zero meaning no membership and 1 standing for full membership. According to Bernhard and Noé, the *N*_α_ C_α_ atoms of the backbone are optimally decomposed into *k* clusters by minimizing the following target function:

$$q\left(c\right)=\overset{k}{\underset{m=1}{\sum }}\overset{N_{\alpha }}{\underset{i=1}{\sum }}\overset{N_{\alpha }}{\underset{j=1}{\sum }}c_{im}c_{jm}D_{ij}=tr\left(c^{T}Dc\right)\to \min$$ (3)

In the formulation of Bernhard and Noé, memberships were assumed to be real numbers. This works successfully in the end but affords tremendous computational expense. In our previous work (Kenn et al., 2014) we were able to improve Bernhard’s and Noé’s method by showing mathematically that the membership coefficients, *c*_*im*_, have in fact to be crisp, i.e., {0,1} . Knowing this in advance drastically speeds up the minimization specified in Eq. 3. Since a given atom can only fully belong to one and only one cluster (no fragmentary membership), optimization can draw on single atom moves between clusters. We applied such a fast random search with single atom moves, followed by exhaustive searches to obtain a global optimum. Each lap of clustering was performed 100000 times and the result with the best target function retained. For computational details, parameter studies and thorough evaluations of accuracy and performance we refer to our previous work (Kenn et al., 2016).

**FIGURE 4:**
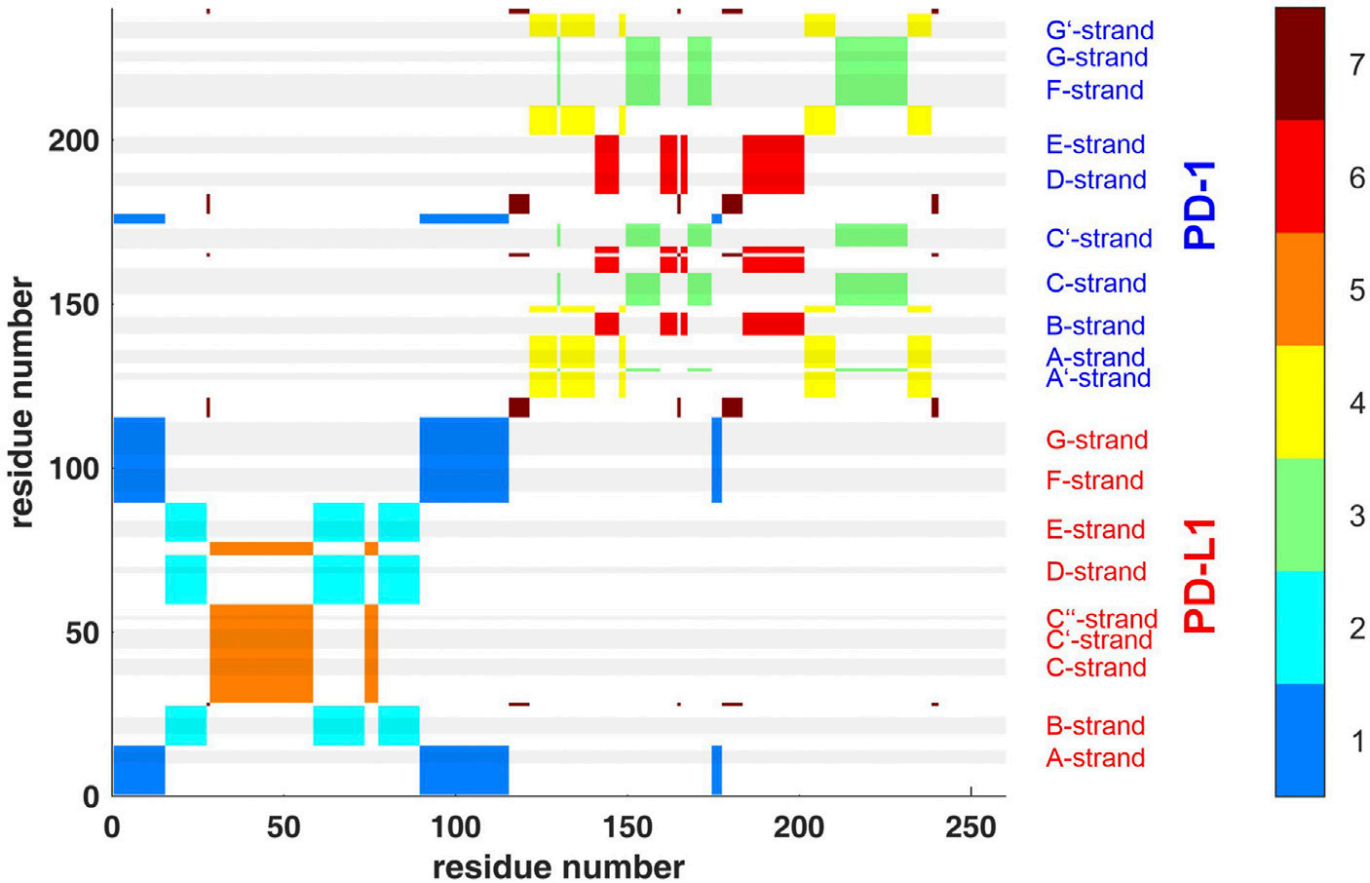
Clustering standard deviations of distance variation (STDDV) with k = 7 over the whole trajectory *t*_1_. The best out of 100000 trials in the search for minimum target function is shown. Each cluster (1–7) is shown in a separate color, see the color bar. The sizes of clusters 1 to 7 were 44, 39, 39, 36, 34, 32 and 16. Elements of secondary structure are indicated by grey shaded bars and corresponding labels.

As a result, spatial clustering yielded crisp memberships, $c_{i,m}^{\left(s\right)}=\left\{0,1\right\}$ , for C_α_-atom *i*, in cluster *m*, within segment *s*, see Figure 4. Note that $1\leq i\leq N_{\alpha }$ , 1≤m≤k and $1\leq s\leq N_{s}$ .

#### 2.3.2 Time-wise consensus clustering

**FIGURE 5:**
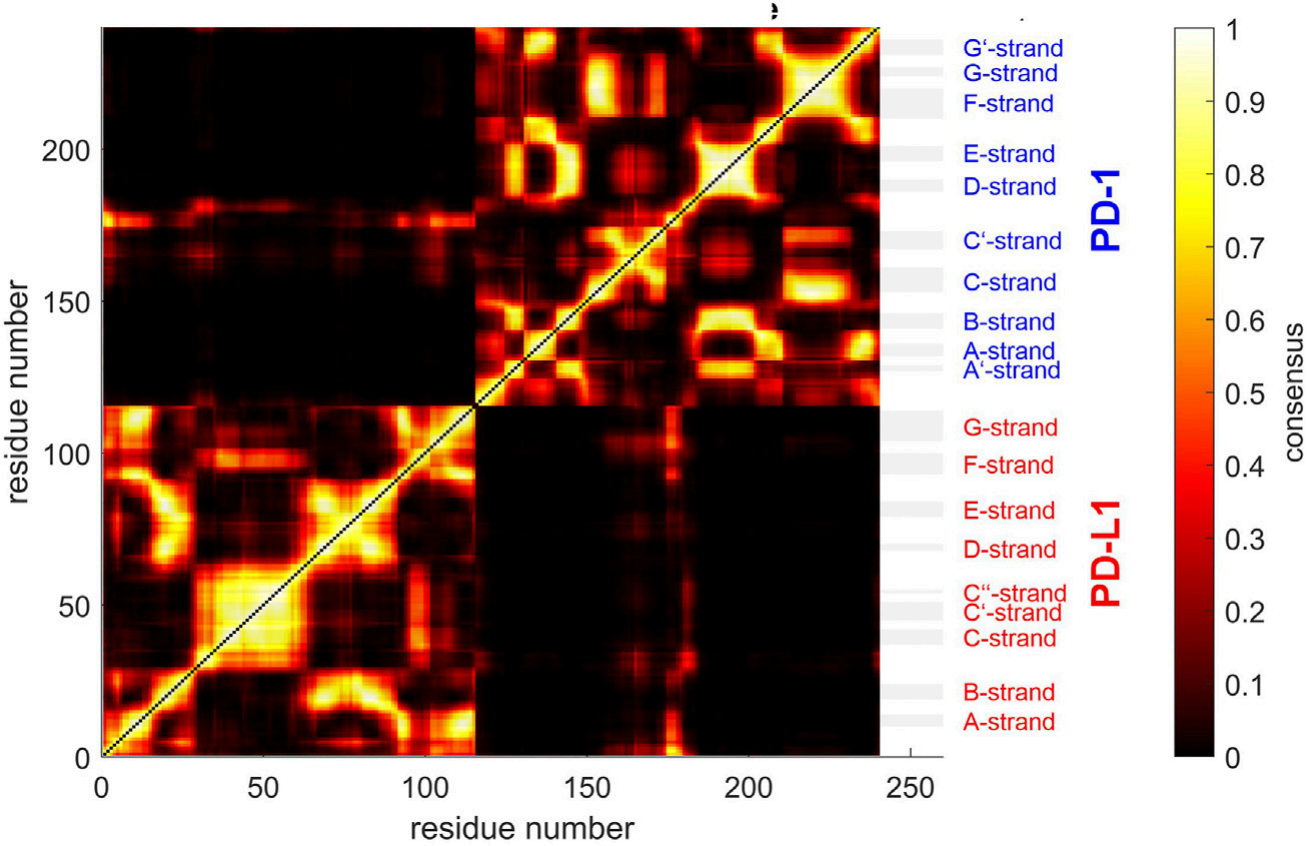
Similarity matrix after temporal consensus clustering trajectory t_1_, shown as scaled color image (SCI). Complex 4ZQK, consisting of receptor PD-1 and PD-L1 as ligand, showing enhanced similarity within two large areas (receptor and ligand, respectively). Note that numbering starts with ligand PD-L1 with residue-ID = 18 (lower left corner), corresponding to residue number *i* = 1 in both axes of the SCI shown. PD-L1 extends over 1 ≤ *i* ≤ 115. The N-terminal end of PD-1 starts with residue-ID = 25 and extends over residues 116 < *i* < 240 towards the right upper corner. Elements of secondary structure are denoted right to the SCI (Zak et al., 2017), with their extensions indicated by horizontal grey shaded bars. Spatial clusters: 7. Note that the number of spatial clusters influences the similarity matrix and is given as input for computation. Consensus (0–500) indicates in how many (out of 500) timewise segments two C_α_ atoms belonged to the same spatial cluster (no matter which cluster that was). Consensus shown normalized to 0–1, see Eq. 5 and color bar.

To arrive at a consensus we start with defining dissimilarity $\Delta_{ij}$ between two C_α_ -atoms *i* and *j* as:

$$\Delta_{ij}=\frac{1}{N_{s}}\overset{N_{s}}{\underset{s=1}{\sum }}\Delta_{ij}^{\left(s\right)}=\frac{1}{N_{s}}\overset{N_{s}}{\underset{s=1}{\sum }}\left(1-\overset{k}{\underset{m=1}{\sum }}c_{im}^{\left(s\right)}\cdot c_{jm}^{\left(s\right)}\right)$$ (4)

with $\Delta_{ij}^{\left(s\right)}=0$ if atoms *i* and *j* belong to the same cluster $C_{m}^{\left(s\right)}$ in segment *s* and $\Delta_{ij}^{\left(s\right)}=1$ otherwise. Summing up $\Delta_{ij}^{\left(s\right)}$ over all segments (*s*) yields the number of segments within which *i* and *j* are not within the same cluster (Monti et al., 2003). Note that the number of segments is an upper bound, e.g. atoms *i* and *j* may reside “not in the same cluster” in 30 segments out of 500. The precise choice of segment length has only minor impact on the results. Shorter segment lengths (e.g., 25 frames per segment) yield a similarity matrix of higher resolution, but entails only minute changes in the final results. Naturally, a minimum length of segments is required to obtain a reliable estimate of variances. Division by the number of segments (*N*_s_) finally renders a normalized measure of dissimilarity between *i* and *j*, relating to the whole trajectory (e.g., 30/500 = 0.06). This dissimilarity lends itself as a proxy for “distance” between atoms in this second lap of clustering (consensus clustering). Since cluster memberships are crisp, $c_{im}^{\left(s\right)}\in \left\{0,1\right\}$ , the concept above can also be expressed more formally (but less intuitively) *via* a product of memberships, see the second part of Eq. 4. Dual to dissimilarity, a *similarity*-matrix can be obtained *via*

$$C_{ij}=1-\Delta_{ij}$$ (5)

see the example displayed in Figure 5. Note that similarity, as defined above, will be used synonymously with “consensus” in the framework of consensus clustering. Naturally, C_α_ atoms in close succession along the backbone appear close to the diagonal and show high consensus, see the color bar.

**FIGURE 6:**
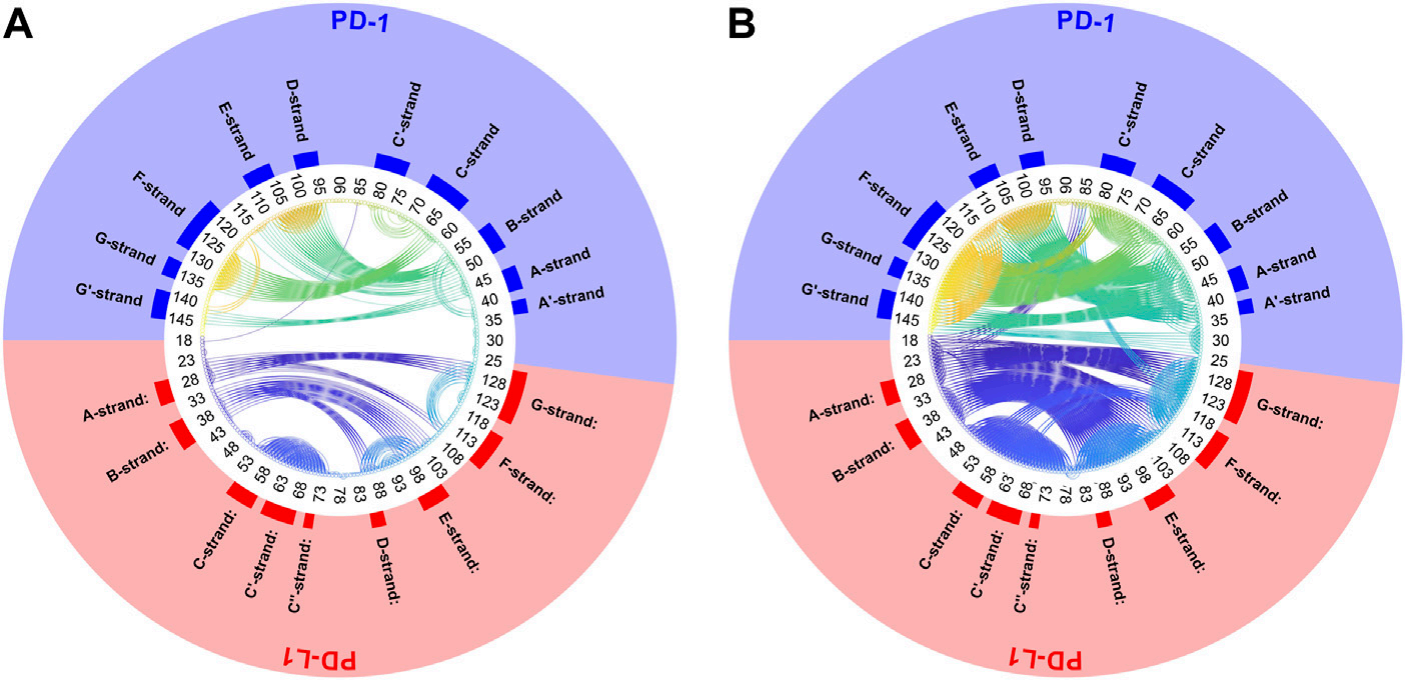
Circular plot of small variations of inter-atom distances for PD-1 complexed with PD-L1. Trajectory 2, spatial clusters *k* = 7. Residues numbered within each chain according to PDB convention. Around the circular plot, elements of secondary structure are indicated. Left: threshold for link to be drawn: $\Delta_{th}=0.04=20/500$ . Right: $\Delta_{th}=0.1=50/500$ .

Another very illustrative way to display consensus between atoms is a circular plot, see Figure 6. All C_α_-atoms are arranged in a circle and a threshold, $\Delta_{th}$ , has to be chosen. Whenever the dissimilarity between two atoms is smaller than the threshold ( $\Delta_{ij}\leq \Delta_{th}$ ), these are connected by a line. Thus, connected atoms show small fluctuation in their distance over time.

#### 2.3.3 Second lap of clustering based on consensus

The dissimilarity matrix $\Delta_{ij}$ was then subjected to agglomerative clustering (Ward, 1963; Jain et al., 1999), evaluating two methods for comparison, “average” and “complete” (Mathworks, 2021). They differ in their mode of linkage, i.e., the way, how the distance between two given (intermediate) clusters is computed: Method “average” takes the mean distance between individuals in different clusters to represent the distance between both clusters. Conversely, method “complete” adopts the largest of those between-cluster distances as the distance between the two clusters.

The methods “average” and “complete” are both appropriate for Euclidean as well as for non-Euclidean distances, which we worked with, after all. A third method (“single”) would also be appropriate for non-Euclidean distances, however it tends to yield a large number of small clusters, what seemed inappropriate for the structure of our molecules. Other methods are restricted to Euclidean distances.

**FIGURE 7:**
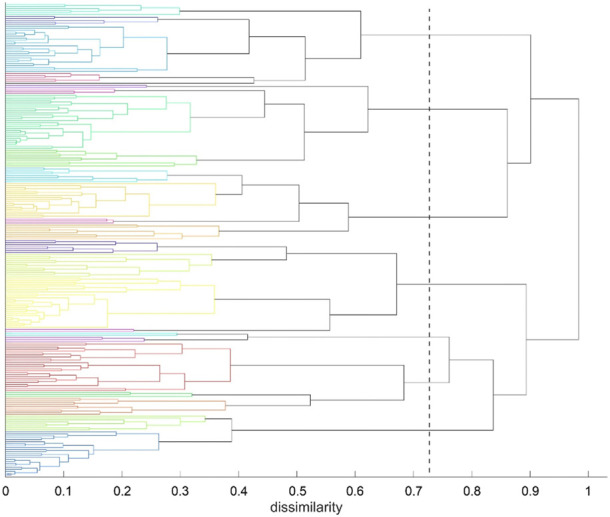
Agglomerative clustering according to inter-atomic time-wise consensus. Spatial clustering (*k* = 7 clusters) within each of 500 time-wise segments. Consensus among these 500 results of clustering was converted into distances and subjected to agglomerative clustering with distance model “average”. For reasons of clarity we call the results of agglomerative clustering “groups” in the following—to distinguish from the results of spatial clustering (“clusters”). Agglomerative clustering was terminated at *N*_G_ = 24 groups. The dashed line indicates *N*_G_ = 7 groups, as an example.

Agglomerative clustering yields a tree-like-structure (dendrogram), an example is shown in Figure 7. At the left vertical axis C_α_ atoms are arranged and colored according to cluster membership, the residue-index being irrelevant here. The horizontal axis shows dissimilarity, in our case values between 0 (each C_α_-atom against itself) and a maximum equal to the number of segments, *N*_s_, into which the trajectory was split (e.g., 500). Note that this maximum applies to the methods chosen in this work but need not apply to other clustering methods, such as “Ward” for example.

Clustering starts at bottom, with each atom representing a cluster of its own (leaves of the tree). Then clustering proceeds upwards (from left to right in Figure 7), in each step joining two clusters, selected among all pairs according to minimum distance. Note that there is no universal definition of “distance” between two clusters but one has to choose among several variants, i.e., “average” or “complete” in this work. Note that “distance” appears on the horizontal axes in Figure 7. As a result, any emerging cluster contains the sum of atoms contained in both of its predecessors. Finally, the algorithm terminates with a cluster containing all atoms, at the root of the tree.

**FIGURE 8:**
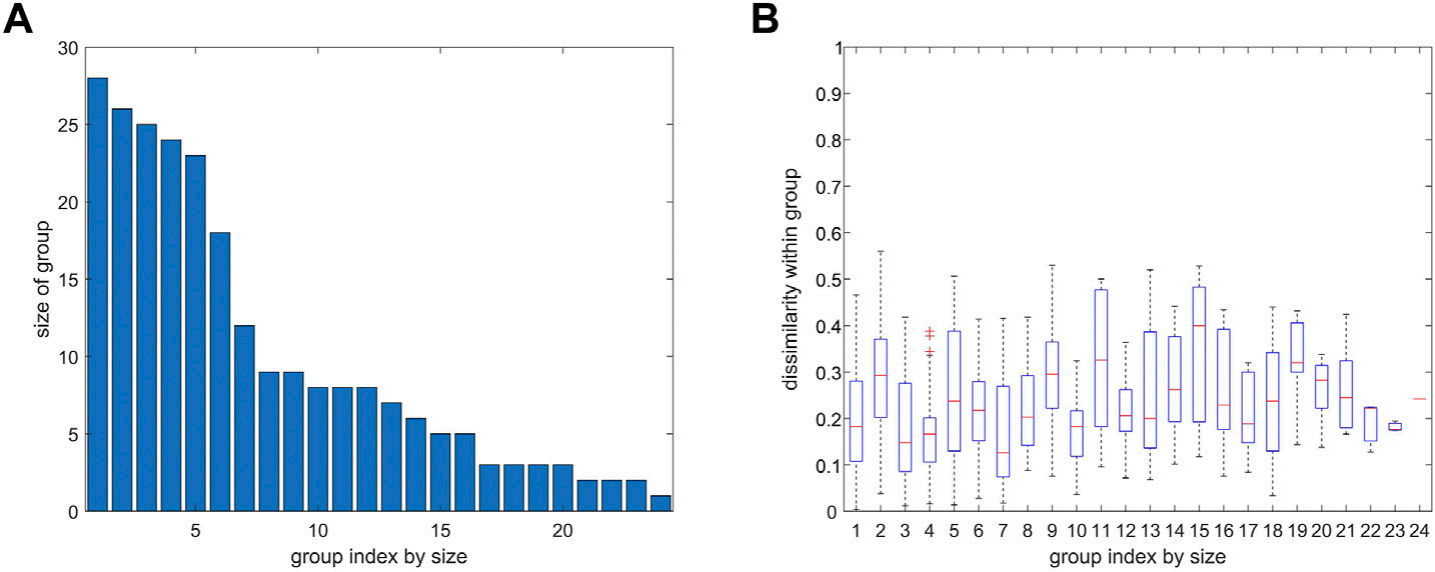
Number of atoms and variability of distance variation within groups from agglomerative clustering. 4ZQK, trajectory *t*_1_, parameters *k* = 7 and *N*_G_ = 24, similar to Figure 7. **(A)** Size of group (number of atoms). **(B)** Homogeneity within groups shown by a boxplot of distance variations between pairs of atoms within each group (mean, quantiles, extremes).

**FIGURE 9:**
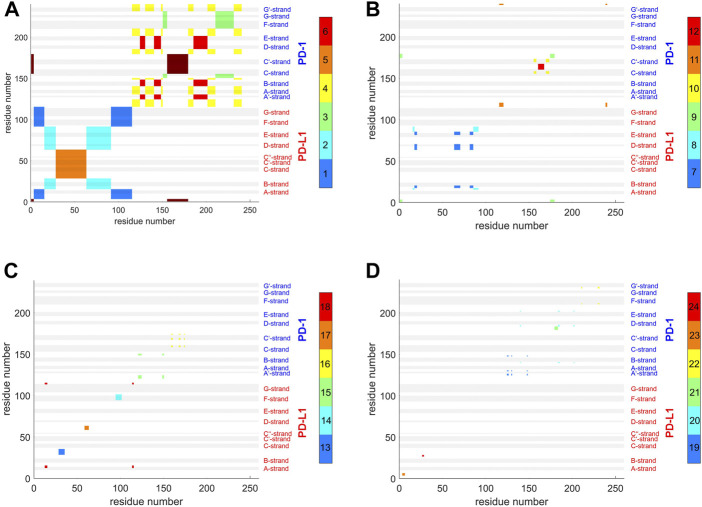
Atom groups resulting from agglomerative clustering consensus. 4ZQK, trajectory *t*_1_, parameters *k* = 7, cutoff *N*_G_ = 24 groups. Note that the groups were internally numbered in order of descending size and each cluster is indicated by the color along the color bar to the right. To visually represent as many as 24 groups, 4 panels were generated for groups 1–6 **(A)**, 7–12 **(B)**, 13–18 **(C)** and 19–24 **(D)**. Note also that each cluster does not need to appear as coherent field in the matrix, since remote atoms in the peptide chain may belong to one and the same cluster, as shown in the circular graph, Figure 6. To identify a single cluster, all fields of the same color within one given panel have to be considered together. All in all, the picture reflects the intricate connections of intra-molecular motions. Elements of secondary structure are indicated by grey shaded bars and corresponding labels.

The tree is then retraced from the root towards the leaves (from right towards left in Figure 7), along decreasing dissimilarity. Whenever a bifurcation is crossed, the number of clusters increases, one by one. One may proceed until a preselected number of clusters, *N*_C_, is encountered (e.g. *N*_C_ = 7 in Figure 7) and thus obtain a corresponding ”cut-point” in terms of dissimilarity, see the dashed line. Quantitatively, the cut-point is computed as the median of those two levels of dissimilarity that have been passed though latest during recovery. In Figure 7, the final cut-point for display was selected at *N*_C_ = 24 groups (left, bottom border of tree). This number of clusters was chosen to accommodate several large, compact domains within the molecule (such as beta-sheets) as well as several smaller parts, such as freely moving loops. This intention has been fulfilled as clearly reflected in Figures 4, 8. These clusters represent a partition of all atoms into a given number (*N*_G_) of groups $\left\{G_{1},G_{2}...G_{N_{G}}\right\}$ , as shown in Figure 9. These groups are shown in different colors.

#### 2.3.4 Estimating the stability of clusters across trajectories

Above we have explained spatial clustering within consecutive segments of a single trajectory and then how to perform agglomerative clustering into domains, based on time-wise stability of these spatial clusters. Resulting clusters were called “semi-rigid”. Finally, we evaluate how much clusters differ between independent trajectories of the same molecular system. This comparison yields an estimate of cluster-stability on an upmost level, and was performed as follows.

For a trajectory *t*, *N*_G_ time-wise consensus clusters $\left\{G_{1}^{\left(t\right)},G_{2}^{\left(t\right)}...G_{N_{G}}^{\left(t\right)}\right\}$ were obtained, with t=1,2,3 , since three trajectories were generated. Let cluster-memberships of atom *i* in cluster *m* within trajectory *t* be denoted by $G_{im}^{\left(t\right)}=\left\{0,1\right\}$ , with $1\leq i\leq N_{\alpha }$ , $1\leq m\leq N_{G}$ and 1≤t≤3 . When comparing results of agglomerative clustering between trajectories, the following problem arises: During agglomeration, emerging labels (identity numbers) of clusters may depend on minute, even somewhat random differences between trajectories. For example, if an existing cluster is to be joined with its “nearest” neighbor cluster, there might be two (or even more) neighbors almost equally “near”. As a consequence, even minute differences between trajectories in such a case induce different decision paths “which cluster wins”, and branch into different joining-operations for each trajectory. Since any new cluster generated (by joining) receives the next available cluster-label in sequence, a certain cluster-label may refer to two physically different groups of atoms in each trajectory. All in all, even though agglomerative clustering may produce nicely compatible physical groups of atoms, the labels of those groups might (and usually will) result totally permuted.

Therefore, after agglomerative clustering two trajectories, a so called “assignment problem” arises (Ramshaw and Tarjan, 2012): How should pairs of corresponding clusters be identified on an algorithmic basis?

**FIGURE 10:**
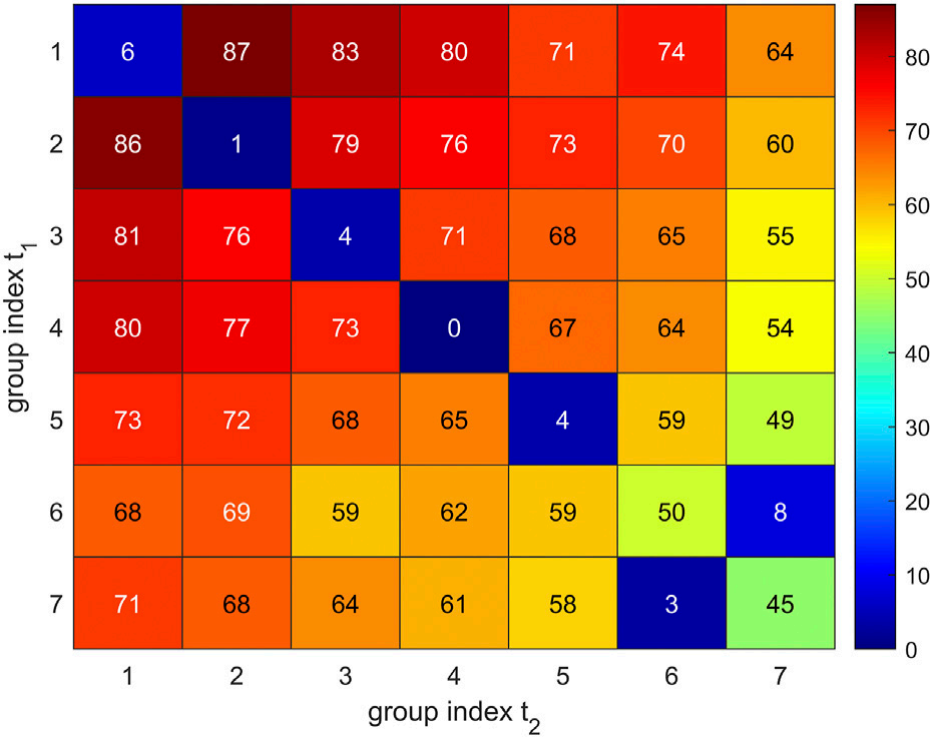
Visual representation of group-matching. Comparison of two sets of 7 **C**_**α**_ groups resulting for trajectory *t*_2_ (horizontal axis) and trajectory *t*_1_ (vertical axis). Group numbers are assigned with decreasing group size (7–1). Values given in matrix elements were evaluated *via* Eq. 6 and represent the loss function $L_{ij}^{\left(t_{1},t_{2}\right)}$ , i.e., the number of **C**_**α**_ atoms not contained in both groups. Low losses indicate good matching between groups and are colored blue, see the color bar. Diagonal elements represent a matching according to group size only, e.g., *L*_*11*_ = 6 indicates that only 7 **C**_**α**_ atoms are not members of these both groups (1–1). Elements off the diagonal represent putative losses if group labels were permuted, e.g., *L*_*12*_ = 87 indicates that 87 **C**_**α**_ atoms would mismatch in a putative comparison between group 1 from *t*_1_ and group 2 from *t*_2_. One can see that for groups 1 to 5, the original labelling (according to group size) is already optimum. Conversely, groups 6 and 7 have to be interchanged for optimum match.

In short, we proceeded as follows: We used the “Hungarian Algorithm”, drawing on the special target function given in Eq. 7. The value given by this target function represents the metric between trajectories. A vivid display is given in Figure 10, including a description how estimates come about for specific groups of C_α_ atoms.

In mathematical detail, the following procedure was performed: For each trajectory, NG (e.g., NG = 24) groups are obtained, and out of NG! possible pairings the best matching has to be determined, labels permuted accordingly, and re-assigned. Only on this basis, a comparison—cluster by cluster—is meaningful.

The assignment problem has been mathematically solved (Kuhn, 1955), based on the “Hungarian algorithm”, was put in a more general frame by Edmunds and Karp (Edmonds and Karp, 1972), and is now available in the MATLAB routine “matchpairs” (Duff and Koster, 2001): The user has to specify a so called “loss function” which quantifies the “loss” compared to a perfect match between two sets of clusters $\left\{G_{1}^{\left(t_{1}\right)},G_{2}^{\left(t_{1}\right)}...G_{N_{G}}^{\left(t_{1}\right)}\right\}$ and $\left\{G_{1}^{\left(t_{2}\right)},G_{2}^{\left(t_{2}\right)}...G_{N_{G}}^{\left(t_{2}\right)}\right\}$ . Note that a comparison is feasible only if both sets contain the same number of clusters, *N*_G_. For example, when evaluating the disparity between a pair of clusters $\left\{G_{i}^{\left(t_{1}\right)},G_{j}^{\left(t_{2}\right)}\right\}$ , one may use the symmetric difference

$$L_{ij}^{\left(t_{1},t_{2}\right)}=\left|\left(G_{i}^{\left(t_{1}\right)}\cup G_{j}^{\left(t_{2}\right)}\right)\backslash \left(G_{i}^{\left(t_{1}\right)}\cap G_{j}^{\left(t_{2}\right)}\right)\right|$$ (6)

as a proxy for a so called loss function, with | | meaning the number of elements in a group (cardinality). If both sets contain the very same atoms, the loss *L* = 0, if they do not share a single atom, the loss $L_{ij}^{\left(t_{1},t_{2}\right)}=\left|G_{i}^{\left(t_{1}\right)}\cup G_{j}^{\left(t_{2}\right)}\right|$ , i.e., it equals the total number of atoms in both groups. For intermediate cases, *L* represents the number of atoms contained in just one of both sets but not in the other (exclusive or-condition).

Solving the assignment problem allows to re-label clusters in a way that clusters with the same index go in pairs (common index *m* replaces *i*, *j*) and this pairing entails minimum overall loss. For this optimum assignment, losses are added over all clusters to obtain the total clustering disparity between both trajectories:

$$D^{\left(t_{1},t_{2}\right)}=\overset{N_{g}}{\underset{m=1}{\sum }}\left|\left(G_{m}^{\left(t_{1}\right)}\cup G_{m}^{\left(t_{2}\right)}\right)\backslash \left(G_{m}^{\left(t_{1}\right)}\cap G_{m}^{\left(t_{2}\right)}\right)\right|$$ (7)

Note that the solution of the assignment problem is not commutative, i.e. $D^{\left(t_{1},t_{2}\right)}\neq D^{\left(t_{2},t_{1}\right)}$ , i.e. it makes a difference in results which trajectory comes first. We shall call it “reference” in the following, e.g. trajectories *t*_2_ and *t*_3_ may be mapped on reference *t*_1._

Optimized re-assignment and joint labelling of clusters allows to boil down each cluster to its “stable kernel”, *K*_m_, made up by those atoms belonging to the “same” cluster in all three trajectories considered:

$$K_{m}^{\left(t_{1},t_{2},t_{3}\right)}=G_{m}^{\left(t_{1}\right)}\cap G_{m}^{\left(t_{2}\right)}\cap G_{m}^{\left(t_{3}\right)},\;m=1,...,N_{G}$$ (8)

Such kernels may be displayed within 3D representations of the molecular complexes.

#### 2.3.5 Relating groups to molecular structures

For each atom *i*, its kernel-membership *k*_*i*_ is known, with $1\leq k_{i}\leq N_{G}$ . This allows for visualization of such groups within 3D representations of the molecular complex. From the memberships we generated Tcl-commands (Welch et al., 2003) to color these groups in VMD (Humphrey et al., 1996), see also the figures shown in the results section.

## 3 Results

### 3.1 Results for whole trajectories

Applying the methods explained above we obtained results for the complex 4ZQK (PD-1 + PD-L1). First, standard deviations *D*_*ij*_ of pair-distances were computed over each whole trajectory, with *N*_f_ = *N*_t_ in Eq. 2. Figure 3 shows the result for trajectories *t*_1_, *t*_2_ and t_3_. Considerable differences between trajectories *t*_1_, *t*_2_ and *t*_3_ can be seen.

Second, spatial clustering was performed over whole trajectories, see an example in Figure 4 for *t*_1_ and k = 7. Note that clustering in any case assigns each atom to one of the clusters, even if its STDDV to quite many other atoms are large, see the conspicuous stripes in shiny yellow in Figure 3. As a consequence, clusters obtained this way inevitably also house atoms not intended to be parts of semi-rigid domains.

### 3.2 Results for segmental clustering

Next, time-wise clustering was performed. Figure 5 shows the similarity matrix with values between 0 and *N*_s_, indicating how often time-wise consensus clustering found two C_α_ atoms within the same cluster. Note that clusters are neither numbered nor labelled in this step, i.e., they do not have unique identifiers related to their “inhabitants” in terms of physical atoms. For example, the pair of C_α,128_ and C_α,237_ may be together in cluster 4 in time-wise segment 129 and together in cluster 5 in time-wise segment 237. This would yield a consistency count of 2 (out of 500). Naturally, the number of segments, *N*_s_, poses an upper limit of consistency, expressing that these two atoms were in the same cluster in all segments.

As consensus relates to linked mobility, most strong linkages were seen within each molecule (chain) of the complex, i.e., within PD-1 and within PD-L1. This resembles the fact that beta-strands cooperatively fold into beta-sheets, and corresponding atoms move in a more concerted way. However, some weaker linkage is also present between both molecules, see the parts in orange for residues of PD-1 towards multiple parts of PD-L1: these regions show consensus. A few C_α_ atoms at the start (i.e., the N-terminal loop) of the ligand even show close relation to this region of PD-1, with consensus around 0.8 (appearing yellow).

Posing a threshold on dissimilarity, e.g., $\Delta_{ij}\leq \Delta_{th}$ , a circular plot can be obtained, see Figure 6. Pairs of atoms are connected by lines to indicate consensus if they appear in different clusters in a fraction of segments smaller than $\Delta_{th}$ . For example, selecting $\Delta_{th}=0.06=30/500$ , connects atoms only if they end up in different clusters in no more than 30 (time-wise) segments, out of 500. Naturally, the larger the dissimilarity threshold, $\Delta_{th}$ , is chosen, the more connection lines populate the circular plot. Moreover, weak similarities, such as those between PD-1 and PD-L1, become visible only if large dissimilarities are tolerated (right panel of Figure 6). They faint away in quite a large percentage of frames.

The above results display concordance (i.e., similarity in movements) between atoms, as it results directly from time-wise consensus clustering, based on pairs of C_α_ atoms. These pairwise results (consensus matrix) were subjected to a further step of analysis, agglomerative clustering, see Figure 7. Note that choosing a certain number of clusters, e.g. *N*_G_ = 24, does not change anything of the algorithm, it just defines the level of cutoff through the tree where splitting into groups is considered as result. Note that dissimilarities between clusters may well exceed the upper limit of dissimilarities $\Delta_{ij}$ between single atoms.

**FIGURE 11:**
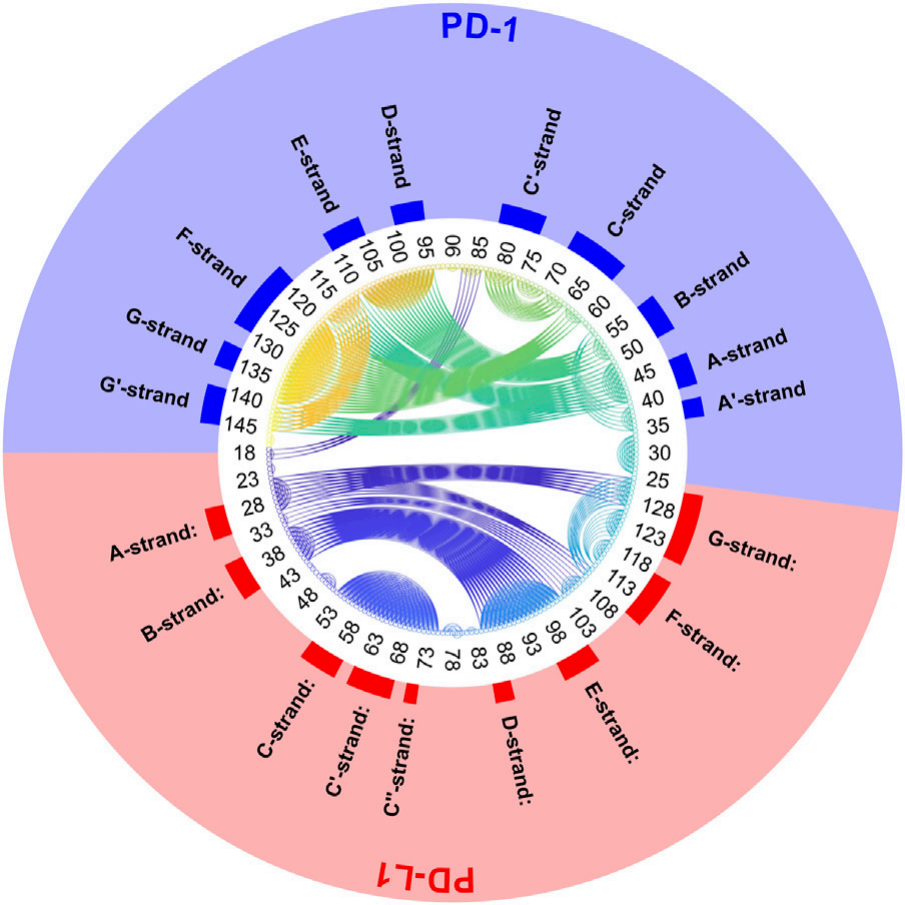
Circular graph of semi-rigid domains from agglomerative clustering time-wise consensus. 4ZQK, trajectory *t*_1_, parameters *k* = 7, cutoff *N*_G_ = 24 groups. Connective lines colored according to C_α_ indices.

Clusters resulting from agglomerative clustering are different in size (number of atoms), see Figure 8. The box plot indicates variability within groups, based in the standard deviations of inter-atom distances used as the key target for spatial clustering. Groups from agglomerative clustering may also be displayed in matrix form, see Figure 9. Like in Figure 5, atoms are numbered consecutively, as they occur in the 4ZQK complex in PDB. Elements of secondary structure have been annotated to hint at possible relations to atomic mobility. In addition, these groups were visualized in circular graphs, see Figure 11.

**FIGURE 12:**
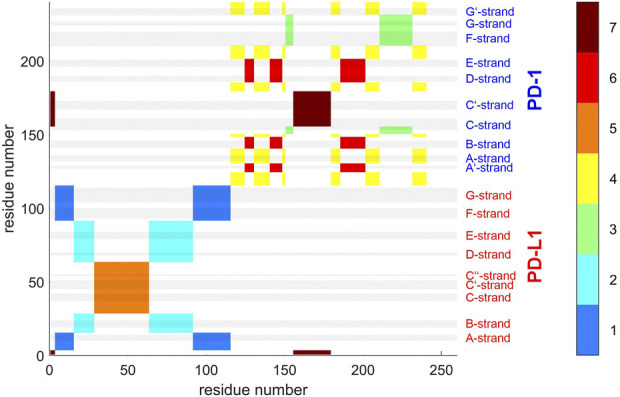
Atom groups for agglomerative clustering with cutoff *N*_G_ = 7 groups. Complex 4ZQK, trajectory *t*_1_, parameters *k* = 7, cutoff *N*_G_ = 7 groups. For easy comparison with Figure 4, each cluster is shown in a separate color, see color bar. Colors were selected to match those of Figure 4, for direct comparison. Elements of secondary structure are indicated by grey shaded bars and corresponding labels.

Agglomerative clustering starts with each atom representing its own cluster and then joins existing clusters. By proceedings upwards level by level, it creates a tree of larger and larger clusters, ending up in one maximum cluster above all others. This tree may be pruned at any level to yield different numbers of clusters. For comparison with clustering STDDV matrices according to Bernhard (Bernhard and Noé, 2010), see Figure 4, we display the agglomerative result pruned at *N*_G_ = 7, see Figure 12. Note that colors have been selected to match those of Figure 4, in order to be directly comparable.

### 3.3 Stability of clusters across trajectories

Note that all visualizations shown so far pertained to one single trajectory and a given set of clustering-parameters (*k*, *N*_G_). It is interesting, however, to evaluate differences in results between trajectories. To these ends we utilized the disparity $D^{\left(t_{1},t_{2}\right)}$ between pairs of trajectories, defined in Eq. 7. Discrepancies in agglomerative clustering between pairs of trajectories were 22, 40 and 28 for (*t*_1_, *t*_*2*_), (*t*_1_, *t*_*3*_) and (*t*_2_, *t*_*3*_), respectively (see Table 3). Comparing three trajectories naturally leads larger discrepancies. For comparison, we also added the results for agglomerative clustering in 7 groups, concordant with the preceding Bernhard-clustering. Note that considering more groups increases the chance for residues to switch between groups, and concomitantly discrepancy increases, however, for generating the final consensus, the more discrepant smaller clusters were disregarded, see below.

**TABLE 3:** Disparity in groups between trajectories. All results refer to *k* = 7 clusters for Bernhard clustering. Agglomerative clustering was performed for 7 and 24 groups, respectively. For each comparison between trajectories, discrepancies in agglomerative clustering are given as numbers of residues within different groups together with corresponding percentages of all residues (240). Note that, for a comparison between three trajectories (right part of table), disparities evaluated according to Eq. 7 depend on the choice of the reference trajectory, listed in position 1—as a coincidence, these results are all equal (44 and 68). Comparing 3 trajectories, means to include differences between 3 pairs of trajectories: For example, an atom counts as disparity if it resides in different clusters for (*t*_1_, *t*_2_) even if it resides in corresponding clusters in (*t*_1_, *t*_3_) and (*t*_2_, *t*_3_). As a consequence, disparities between triples of trajectories appear larger than those between pairs.

Groups	Trajectory comparison					
	(*t*_1_, *t*_2_)	(*t*_1_, *t*_3_)	(*t*_2_, *t*_3_)	(*t*_1_, *t*_2_, *t*_3_)	(*t*_2_, *t*_1_, *t*_3_)	(*t*_3_, *t*_1_, *t*_2_)
7	40 (16.7%)	28 (11.7%)	22 (9.2%)	44 (18.3%)	44 (18.3%)	44 (18.3%)
24	52 (21.7%)	44 (18.3%)	41 (17.1%)	68 (28.3%)	68 (28.3%)	68 (28.3%)

**FIGURE 13:**
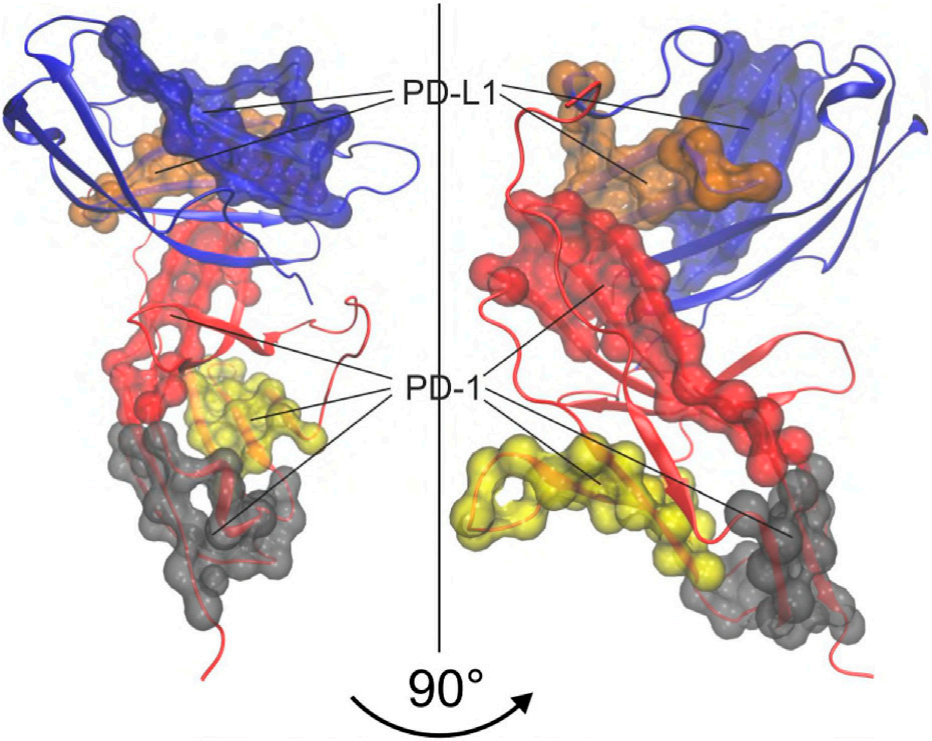
3D-Visualization of semi-rigid domains resulting from spatio-temporal consensus clustering and consensus over trajectories. Groups obtained from agglomerative clustering were consolidated over 3 trajectories to obtain “kernels”, representing semi-rigid domains. The 5 largest kernels are colored and shown as surface representations. Note that kernels in red, grey and yellow belong to PD-1 while kernels in blue and ochre belong to PD-L1.

Finally, however, we also created a consensus between trajectories by estimating “kernels” of atoms belonging to the same cluster in all three trajectories, see Eq. 8. Note that the labelling of agglomerative groups originally varies randomly between trajectories and has to be consolidated as described in the methods section. Such a consolidated numbering—and a corresponding coloring—was used to outline the kernels within a 3D model of the molecular complex, see Figure 13. These kernels are considered as the “semi-rigid domains” aimed at.

## 4 Discussion

We applied the method of spatio-temporal clustering to the PD-1/PD-L1 complex, aiming at identifying semi-rigid domains within these molecules. Such domains are considered a highly important basis for coming computational research since any detection of minute movement patterns requires to fit molecular configurations to stable kernels. Minute and interesting movement patterns, e.g., of active loops, may then be characterized with reference to such kernels.

During the course of an MD-simulation, larger portions (“domains”) of a molecule might collectively move slowly but move broadly back and forth in amplitude. Inside such a domain, however, single amino acids and even more single atoms oscillate at much higher frequencies. The goal is to separate these two types of movement occurring on different spatial and time scales: semi-rigid domains as a whole should go along with the larger but slower movements, while housing those many tiny oscillations of their “inhabitant“ atoms. As a result, a single atom performs both motions in superposition—small oscillations at high frequency, superimposed on larger and much slower collective motions of its corresponding domain. Both types of motion in combination influence the distances to its neighbor atoms.

In a non-supervised approach, one can only draw on the variation of distances as such, without knowing their origin (tiny oscillations of single atoms or large-scale movement of whole domains). Clustering atoms with respect to variations of pair-distances will therefore yield different results (clusters), when performed on different (time-wise) parts of a trajectory. Finally, however, a smart clustering algorithm should yield larger clusters “moving” in accordance with those larger domains, each of these holding much the same groups of atoms as inhabitants (members) over time.

For a start, we computed the standard deviations of distance variations (STDDV) matrices of whole trajectories (Figure 3). Since these matrices did not reveal prominent structures which could be clustered right away, we adopted a refined, three-step procedure. Moreover, distinct differences between trajectories became apparent in these matrices. We have to conclude that the system obviously inhabited different configurational sub-spaces in each trajectory, and simulation time has to be extended in coming studies to closer approach ergodicity and visit all portions of configuration space appropriately.

In the present work, matrices with different properties were studied. The STDDV-matrix does not reflect distances as such but rather variations in distance and therefore in general will not fulfil the triangle inequality. Incidentally, the Bernhard algorithm does not require fulfillment of the triangle inequality. In the second step of our algorithm we computed the dissimilarity matrix, Eq. 4, which fulfills the triangle inequality. This was a main reason for us to adopt this multi-step procedure.

To refine clustering we adopted a three-step procedure: First, clustering according to distance variation, but separately over short segments of the trajectory. Second, these results were consolidated over all segments of the trajectory by characterizing consensus for each pair of atoms: the percentage of time-wise segments in which these two atoms shared (resided in) the same cluster. Note that this second step yielded but pairwise information (consensus matrix), visualized in various forms (Figures 5, 6). Third, we performed agglomerative clustering to derive domain-like regions of coherence, the final result, shown in Figure 13. Note that cluster memberships after agglomerative clustering are in general different from those obtained by spatial clustering in the first lap.

The most intuitive approach would have been agglomerative hierarchical clustering, (Kaufman and Rousseeuw, 1990; Teukolsky et al., 2007). In a preliminary examination of the STDDV matrices (Figure 3) we found that an important precondition of agglomerative clustering is only poorly satisfied by MD data: Atoms may switch between clusters quite freely, without severely changing the target-function (minimum distance variability within clusters). This may easily deteriorate agglomerative clustering, and therefore we refrained from it as a first step. However, in future studies it would be interesting to mend this drawback, possibly by selecting more sophisticated models for linkage between clusters (others than “average” or “complete”). Also, agglomerative clustering allows to optimize the cut-off (i.e. the number of groups, *N*_G_) according to formal criterions such as consistency (Mathworks, 2021). Linkage and cut-off could be systematically evaluated and optimized.

The achievement of the present work is the unsupervised consolidation of quite large domains within the molecular complex, despite considerable movements of its member atoms. Results were additionally consolidated by repeating the entire analysis for three independent trajectories and considering the overlap between these three replicates of a cluster as the final, reliable rigid domain. Based on these semi-rigid domains, subtle movements of active regions may be evaluated in future studies, scrutinizing the molecular basis of receptor activation and action of drugs, including checkpoint blockers in oncology.

## Data Availability

Publicly available datasets were analyzed in this study. This data can be found here: https://www.rcsb.org/structure/4ZQK, https://www.rcsb.org/structure/5GGS.
